# Influence of opacity and curing distance on the microhardness and bottom-to-top hardness ratio of an APS-based nanohybrid resin composite

**DOI:** 10.4317/jced.64586

**Published:** 2026-09-15

**Authors:** Ezgi Erden-Kayalidere, Sadettin Arik, Burak Ahmet Deveci, Merve Kocuk

**Affiliations:** 1 Istanbul Kent University, Faculty of Dentistry, Department of Restorative Dentistry, Istanbul, Turkey; 2 Dentist, Private Practice, Istanbul, Turkey

**Keywords:** APS-based composite, curing distance, microhardness, opacity, polymerization efficiency, resin composite.

## Abstract

**Background:**

To evaluate the effects of manufacturer-designated opacity category (enamel, body and dentin) and curing distance on the top and bottom surface microhardness and the bottom-to-top hardness ratio of an APS-based nanohybrid resin composite.

**Materials and Methods:**

Forty-two disc-shaped specimens (2 mm thick, 8 mm in diameter) were prepared from an APS-based nanohybrid resin composite in three manufacturer-designated opacity categories (enamel WE, body A1, dentin DA1), each divided according to curing distance (0 or 2 mm from the light guide tip). Polymerization used an LED light-curing unit at 1200 mW/cm^2^ for 20 s. After 24 h in distilled water at 37°C, Vickers microhardness was measured on both surfaces and the bottom-to-top ratio was calculated as an indirect indicator of curing adequacy. Data were analyzed by two-way ANOVA with appropriate post-hoc comparisons (p<0.05).

**Results:**

Opacity category significantly affected top surface microhardness, bottom surface microhardness, and the bottom-to-top microhardness ratio (p<0.001). Bottom surface microhardness was also significantly influenced by curing distance (p=0.003). Enamel WE was the only category that exceeded the commonly used 80% hardness ratio threshold at both curing distances, whereas body A1 and dentin DA1 remained below this threshold. No significant opacity category × curing distance interaction was detected for any of the evaluated parameters.

**Conclusions:**

Within the limitations of this in vitro study, manufacturer-designated opacity category significantly influenced the hardness-based curing outcomes of the tested composite. Enamel WE showed the highest bottom-to-top hardness ratio and was the only category to exceed the 80% criterion at both curing distances. Increasing the curing distance significantly reduced bottom surface microhardness but did not significantly affect the hardness ratio. The manufacturer-designated opacity categories of this composite should not be assumed to exhibit equivalent hardness-based curing outcomes under an identical light-curing protocol.

## Introduction

Resin-based composites are among the most frequently used restorative materials in contemporary dentistry because of their favorable esthetic properties, minimally invasive application protocols, and continuously improved physical and mechanical behavior. Their clinical performance, however, is strongly dependent on adequate light polymerization. Insufficient polymerization may result in reduced surface hardness, lower wear resistance, impaired marginal integrity, increased water sorption, color instability, and greater residual monomer release, all of which may compromise the longevity and biological safety of the restoration [1-4]. 

Light-curing effectiveness is influenced by several interrelated variables, including the irradiance and emission spectrum of the light-curing unit, exposure time, light-tip position, distance from the restoration surface, increment thickness, and the optical and chemical characteristics of the restorative material [4-7]. In daily practice, the curing light may not always be positioned in direct contact with the composite surface, and the restoration may contain shades with different levels of opacity and translucency. 

The optical behavior of resin composites is largely determined by the interaction between the organic resin matrix, inorganic filler particles, pigments, opacifiers, and the photoinitiator system. Light transmission through a composite material can be reduced by absorption and scattering, especially when there is a mismatch between the refractive indices of the resin matrix and filler phase [8,9]. More opaque or chromatic shades may absorb and scatter more curing light than translucent shades, reducing light penetration and potentially impairing polymerization at the bottom surface. Recent studies have confirmed that shade and increment thickness significantly affect light transmission and microhardness in contemporary resin composites [10,11]. 

In esthetic restorative procedures, resin composites are commonly supplied in enamel, body, and dentin opacities to reproduce the optical stratification of natural teeth. In multilayered restorative systems, enamel shades are generally designed to provide greater translucency, whereas dentin shades are typically formulated to provide greater masking ability and color saturation; however, the actual optical properties are material-dependent. Barata Mills *et al*. recently evaluated the translucency and polymerization ability of contemporary resin composites and highlighted that enamel, body, and dentin shades may differ in both optical behavior and curing potential [12].

The distance between the light-curing tip and the composite surface is another clinically relevant factor affecting polymerization behavior. Ideally, the light guide should be positioned in contact with and perpendicular to the restoration. However, this is not always achievable because of cavity configuration, cusp height, proximal boxes, matrix bands, limited mouth opening, or posterior access limitations. As the distance increases, the irradiance reaching the restoration surface may decrease, and the effective radiant exposure delivered to the material may be reduced [4,5]. Hasanain *et al*. demonstrated that increased light-curing distance negatively affected microhardness profiles of resin composites, especially at deeper regions [13]. 

Photoinitiator chemistry is another determinant of polymerization behavior. Camphorquinone remains the most widely used photoinitiator in resin composites because of its compatibility with blue-light LED curing units. However, its intrinsic yellow color may affect the final shade of highly esthetic restorations. To overcome this limitation, alternative or modified photoinitiator systems have been introduced. The Advanced Polymerization System (APS) was developed to reduce the camphorquinone-related yellowing effect while maintaining adequate polymerization performance. Tapety *et al*. reported that APS-containing composites may exhibit degree of conversion and mechanical properties comparable to those of materials containing conventional photoinitiator systems, depending on the specific material formulation and curing conditions [14]. 

The influence of composite shade and light-curing distance on surface hardness is already established for conventional camphorquinone-based materials. Thome *et al*. reported that both curing-tip distance and shade affected the Vickers hardness of microhybrid and nanofilled composites [15]; Hasanain *et al*. showed that increasing the curing distance reduced microhardness in the deeper regions of bulk-fill composites [13]; and Barcelos *et al*. demonstrated that manufacturer-recommended exposure times do not guarantee equivalent through-thickness polymerization across materials [16]. The general effects of shade and curing distance are therefore not in question, and the present study does not claim to establish them. What has not been reported is how these factors are associated with hardness-based curing outcomes within an APS-based system designed to reduce camphorquinone-related yellowing while maintaining polymerization performance. To our knowledge, no study has compared the manufacturer-designated enamel, body and dentin opacity categories of a single APS-based composite at two curing distances (0 and 2 mm). Thus, the novelty of the present study lies in the simultaneous evaluation of manufacturer-designated opacity category and curing distance within a single APS-based composite. Accordingly, the aim of this *in vitro* study was to evaluate the effects of manufacturer-designated opacity category (enamel WE, body A1 and dentin DA1) and curing distance (0 and 2 mm) on top surface microhardness, bottom surface microhardness, and the bottom-to-top microhardness ratio of an APS-based nanohybrid resin composite.

The null hypotheses were as follows: First, manufacturer-designated opacity category would have no statistically significant effect on top surface microhardness, bottom surface microhardness, or the bottom-to-top microhardness ratio; second, curing distance would have no statistically significant effect on these parameters; and third, the interaction between opacity category and curing distance would have no statistically significant effect on top surface microhardness, bottom surface microhardness, or the bottom-to-top microhardness ratio.

## Materials and Methods

*Study overview*

This *in vitro* study evaluated the effects of manufacturer-designated opacity category and curing distance on the top and bottom surface microhardness of an APS-based nanohybrid resin composite (Elora APS). The three categories evaluated (enamel WE, body A1 and dentin DA1) correspond to the manufacturer's opacity designations and were therefore analyzed as a categorical factor rather than as quantitatively measured optical values. Specimens were prepared at a standardized thickness of 2 mm and polymerized using an LED curing unit at two light-tip distances (0 mm and 2 mm). Vickers microhardness was measured on both surfaces, and the bottom-to-top microhardness ratio was calculated as an indirect indicator of curing adequacy through the specimen thickness.

*Sample size calculation*

A sample size calculation was performed using G*Power 3.1.9.7 software (Heinrich-Heine-Universität Düsseldorf). The bottom-to-top microhardness ratio (bottom/top × 100) was defined as the primary outcome. Assuming a large effect size (*f*=0.50, according to Cohen's conventions), *α*=0.05 and 80% power, and using the F test family "ANOVA: Fixed effects, special, main effects and interactions" with six groups and a numerator df of 2 for the interaction, a total of 42 specimens was required for the 3×2 factorial design, corresponding to n=7 per group.

*Specimen preparation*

Resin composite specimens were prepared using polytetrafluoroethylene (PTFE) molds (2 mm thickness × 8 mm diameter). An Elora APS (FGM, Joinville-SC, Brazil) resin composite (Table 1) was placed into the molds in a single increment and covered with a transparent plastic matrix strip (Mylar Strip, SS White) and a 1 mm thick glass slide. Gentle pressure was applied to obtain a standardized flat surface. After removal of the glass slide, the transparent matrix strip remained in place during light curing for all specimens. The curing protocol differed only with respect to the distance between the light guide tip and the specimen surface, according to group allocation.

A total of 42 specimens were fabricated and allocated into three manufacturer-designated opacity categories (n=14 per group): Enamel (WE), body (A1) and dentin (DA1). Each opacity category was further subdivided into two subgroups (n=7) according to curing distance: Direct contact with the light guide tip (0 mm) and 2 mm distance. For the 2 mm groups, an additional identical PTFE mold was positioned over the specimen mold to standardize the separation between the light guide tip and the composite surface.

Polymerization was performed using an LED light-curing unit (Bluephase PowerCure, Ivoclar Vivadent, Schaan, Liechtenstein) in high-power mode (390-500 nm) at an irradiance of 1200 mW/cm² for 20 s. Following polymerization, all specimens were stored in distilled water in an incubator at 37 °C for 24 h before microhardness testing. The overall experimental design is summarized in Figure 1.

*Measurement of microhardness*

Surface microhardness was measured on both the top and bottom surfaces of each specimen using a Vickers microhardness tester (Falcon 400, Innovatest, The Netherlands). Three indentations were performed on each surface using a load of 300 g applied for 15 s. The indentations were positioned at least 1 mm apart from each other and from the specimen margins to avoid interaction between indentations. The diagonal lengths of the indentations were measured, and the Vickers hardness number (VHN) was automatically calculated by the device software. The mean of the three measurements was recorded as the VHN for each surface.

The bottom-to-top microhardness ratio (HR) was calculated using the following formula:

HR (%) = (VHN_bottom_ / VHN_top_) × 100.

A bottom-to-top microhardness ratio of 80% or higher has commonly been used as an indirect criterion for adequate curing through the specimen thickness [17].

*Statistical analyses*

Statistical analysis was performed using IBM SPSS Statistics software (version 30.0). The normality of the data distribution was assessed by examining the skewness and kurtosis values. Since these values for the top and bottom surface microhardness measurements, as well as the hardness ratios, were found to be within the range of -1.5 to +1.5, the data were considered to be normally distributed. Homogeneity of variances across the six experimental groups was verified using Levene's test based on the median, and the assumption was satisfied for all three outcomes (top surface microhardness, *p*=0.894; bottom surface microhardness, *p*=0.643; hardness ratio, *p*=0.961).

Two-way ANOVA was used to determine the main effects of opacity category and curing distance, and their interaction, on top surface microhardness, bottom surface microhardness and the bottom-to-top hardness ratio. Simple main effects were then examined using the error term of the two-way model. Differences among the three opacity categories within each curing distance were tested with the Tukey HSD post-hoc test; because curing distance had only two levels, the comparison between 0 and 2 mm within each opacity category was made as a single pairwise contrast against the same error term, for which no multiplicity adjustment is required. Because the interaction was not significant for any outcome, the three opacity categories were additionally compared on their marginal means averaged across curing distances (Tukey HSD, n=14 per category, using the same error term); these marginal comparisons are the ones reported in the text, whereas the within-distance comparisons (n=7) are indicated by the lowercase letters in Table 2. All analyses were performed at a significance level of *p*<0.05.

## Results

*Microhardness evaluation (top and bottom surfaces)*

Top surface microhardness, bottom surface microhardness and the bottom-to-top hardness ratios of the six experimental groups are presented in Table 2, and the corresponding two-way ANOVA in Table 3. Top surface microhardness was significantly affected by opacity category (*p*<0.001, ηp²=0.870), whereas neither curing distance (*p*=0.071) nor the opacity category × curing distance interaction (*p*=0.510) was significant. Averaged across curing distances, body A1 (90.01 ± 2.23 VHN) and dentin DA1 (89.80 ± 2.19 VHN) showed significantly higher top surface microhardness than enamel WE (79.20 ± 2.04 VHN; *p*<0.001) and did not differ from each other (*p*=0.959).

Bottom surface microhardness was significantly affected by both opacity category (*p*<0.001, ηp²=0.732) and curing distance (*p*=0.003, ηp²=0.219), whereas the opacity category × curing distance interaction was not significant (*p*=0.200). Averaged across curing distances, dentin DA1 (59.44 ± 3.76 VHN) had significantly lower bottom surface microhardness than enamel WE (70.47 ± 2.43 VHN) and body A1 (69.08 ± 4.36 VHN; *p*<0.001), which did not differ from each other (*p*=0.493). Averaged across opacity categories, bottom surface microhardness was lower at 2 mm (64.76 ± 5.82 VHN) than at 0 mm (67.90 ± 6.08 VHN), consistent with the significant main effect of curing distance. Within individual opacity categories, this reduction reached significance only for body A1 (71.75 ± 4.50 VHN at 0 mm versus 66.41 ± 2.10 VHN at 2 mm; F(1,36)=9.71, *p*=0.004), and not for enamel WE (*p*=0.604) or dentin DA1 (*p*=0.071). The lowest group mean was recorded for dentin DA1 at 2 mm (57.85 ± 3.02 VHN).

*Bottom-to-top hardness ratio*

The bottom-to-top hardness ratio, used as an indirect indicator of curing adequacy through the specimen thickness, was significantly affected by opacity category (*p*<0.001, ηp²=0.847), whereas neither curing distance (*p*=0.078) nor the opacity category × curing distance interaction (*p*=0.133) was significant. Averaged across curing distances, all three opacity categories differed significantly from one another (*p*<0.001): Enamel WE gave the highest ratio (89.04 ± 4.11%), followed by body A1 (76.79 ± 5.22%) and dentin DA1 (66.21 ± 4.14%). Enamel WE was the only category to exceed the commonly used 80% threshold, and did so at both curing distances (88.61 ± 3.60% at 0 mm and 89.47 ± 4.82% at 2 mm), whereas body A1 (79.70 ± 5.41% and 73.88 ± 3.17%) and dentin DA1 (67.32 ± 4.26% and 65.10 ± 4.01%) remained below it. Although the main effect of curing distance on the hardness ratio was not significant, the within-category reduction was significant for body A1 (F(1,36)=6.48, *p*=0.015) but not for enamel WE (*p*=0.710) or dentin DA1 (*p*=0.337).

## Discussion

The present study investigated the influence of opacity category and curing distance on hardness-based indicators of curing adequacy in an APS-based nanohybrid resin composite, namely the top and bottom surface microhardness and the bottom-to-top hardness ratio. Based on the findings, the first null hypothesis was rejected, since opacity category significantly affected top surface microhardness, bottom surface microhardness, and hardness ratio. The second null hypothesis was partially rejected, as curing distance significantly influenced bottom surface microhardness but did not significantly affect top surface hardness or the overall hardness ratio. The third null hypothesis was not rejected, because no significant interaction between opacity category and curing distance was detected for any of the evaluated parameters.

Previous investigations have demonstrated that APS-containing resin composites can achieve physicochemical and mechanical properties comparable with those of conventional camphorquinone-based materials under standardized curing conditions [16,18,19]. Tapety *et al*. evaluated a commercial composite containing APS technology and reported that this system allowed a reduction in camphorquinone concentration without impairing physicochemical properties; the APS-containing material showed degree of conversion and mechanical properties comparable to those of conventional resin composites [14]. Similarly, Dressano *et al*. showed that reduced concentrations of camphorquinone within a ternary initiator system may enhance polymerization-related properties in dental resin composites, indicating that modified initiator strategies can improve material performance when the initiator balance and curing conditions are properly optimized [20]. 

Among the evaluated parameters, the bottom-to-top hardness ratio provided information on through-thickness curing rather than on the hardness of the irradiated surface alone. A bottom-to-top hardness ratio of at least 80% has commonly been used as an indirect criterion for adequate curing through the restoration thickness [3,21]. In the present study, only the enamel opacity exceeded this threshold at both curing distances, whereas the body opacity remained slightly below the threshold at 0 mm and decreased further at 2 mm. The dentin opacity consistently demonstrated the lowest hardness ratio values under both curing conditions. 

Interestingly, the enamel opacity exhibited the highest hardness ratio despite presenting lower top surface microhardness than the body and dentin opacities. A plausible explanation is that the enamel opacity may transmit light more efficiently to the deeper regions of the specimen, producing a more homogeneous curing pattern, whereas the body and dentin opacities may attenuate the incident light to a greater extent and therefore reach lower bottom-to-top hardness ratios; in absolute terms only the dentin opacity had a significantly lower bottom surface microhardness than the enamel opacity. This interpretation must be regarded as a hypothesis rather than a demonstrated mechanism, because no optical data are reported in the present study and the enamel, body and dentin categories are manufacturer designations that may also differ in pigment composition and filler-related light scattering rather than in translucency alone. Confirming it would require optical characterization of each opacity to be evaluated alongside the hardness outcomes. 

The influence of opacity observed in the present study is consistent with the optical behavior of contemporary resin composites reported in the literature, although this consistency is inferred from optical data published in the literature rather than from optical results presented in this manuscript. Light propagation within resin-based materials is affected by several factors, including translucency, filler content, filler size and distribution, pigment concentration, and the refractive index mismatch between the organic matrix and inorganic fillers [10,11]. In that literature, increased opacity is reported to promote greater light scattering and absorption, thereby reducing the radiant energy reaching deeper portions of the restoration; whether the three manufacturer-designated categories tested here follow such an ordering was not determined in the present study. 

The present findings are in agreement with those reported by Wang *et al*., who demonstrated that the effect of curing light beam characteristics on composite microhardness is influenced by both shade and specimen thickness, emphasizing the importance of optical properties when evaluating polymerization behavior [11]. Likewise, Heyder *et al*. reported that shade and increment thickness significantly affect light transmission through contemporary nanofilled and nanohybrid resin composites, with dentin shades generally exhibiting lower light transmission than more translucent shades [10]. The present study adds to these observations the specific finding that, within one APS-based composite developed to optimize polymerization performance, the manufacturer's opacity categories were still associated with different through-thickness curing.

One aspect that should be considered when interpreting the present findings is that all opacity categories were polymerized using a standardized exposure time of 20 s, although the manufacturer recommends a longer curing time (30 s) for the dentin opacity. Therefore, the lower bottom surface microhardness and hardness ratio observed for the dentin opacity may have been influenced, at least partially, by the shorter exposure time used in the present study. However, this explanation alone is unlikely to account for the observed differences, since the body opacity, for which the manufacturer also recommends a 20 s curing time, likewise failed to achieve the commonly used 80% hardness ratio threshold. Besides opacity category, curing distance also affected bottom surface microhardness, although its influence was less pronounced. Increasing the curing distance from 0 mm to 2 mm significantly reduced bottom surface microhardness but did not significantly affect either top surface microhardness or the bottom-to-top hardness ratio. 

The reduction in bottom surface microhardness observed at 2 mm is consistent with the well-established relationship between irradiance and the distance separating the light-curing unit from the restorative material [5,13,22,23]. As the light guide moves away from the restoration surface, beam divergence and energy attenuation reduce the amount of radiant energy delivered to deeper portions of the composite. Because ideal contact between the light guide and the restoration is often prevented by cusp morphology, matrix systems or limited access, even small increases in curing distance may affect polymerization at the bottom of the restoration [22,24-26]. 

These observations agree with the findings of Hasanain *et al*., who demonstrated that increasing the distance between the light-curing unit and resin composite significantly reduced microhardness values, particularly in deeper regions of the material [13]. Likewise, Rueggeberg *et al*. emphasized that polymerization effectiveness depends not only on the nominal irradiance of the curing unit but also on factors such as light-tip positioning, beam profile, exposure time, and the actual radiant energy delivered to the restoration [4]. 

No statistically significant interaction between opacity category and curing distance was detected for any outcome, indicating that the present study did not provide evidence that the effect of curing distance differed among the opacity categories within the tested range. It is also possible that the nominal irradiance of the high-power LED unit (1200 mW/cm² for 20 s) partially compensated for the energy loss associated with the 2 mm curing distance, thereby minimizing its effect on the bottom-to-top hardness ratio.

From a clinical perspective, the present findings demonstrate that opacity category selection should not be regarded solely as an esthetic consideration but also as an important factor influencing hardness-based curing outcomes. In restorative procedures requiring body and dentin opacities to reproduce the optical characteristics of natural teeth or to enhance masking ability, clinicians should be aware that these opacities may achieve a lower bottom-to-top hardness ratio than enamel opacities when identical curing protocols are used, and that the dentin opacity in particular reached a significantly lower bottom surface microhardness. Therefore, when polymerizing body and dentin layers of this material, careful attention should be paid to curing distance, exposure time and the manufacturer's curing recommendations. These implications are material-specific and apply only to the tested APS-based composite; they should not be generalized to APS-based composites as a class.

Several limitations should be acknowledged. First, only one commercial APS-based nanohybrid resin composite was tested, so the findings characterize this product and cannot be generalized to APS technology as a whole or to other photoinitiator systems. Second, the curing-distance range was narrow, comprising only 0 and 2 mm at a single increment thickness of 2 mm; clinically more compromised situations involving greater distances, angulation of the light guide, shadowing by adjacent structures or reduced irradiance were not simulated and may produce larger differences. Third, the bottom-to-top microhardness ratio is an indirect surrogate for the degree of conversion; spectroscopic measurement by Fourier-transform infrared or Raman spectroscopy would provide stronger and more direct evidence of polymerization. Fourth, all specimens were evaluated 24 h after polymerization, and no thermocycling, water aging, fatigue, wear or other long-term mechanical testing was performed, so it is not known whether the differences observed immediately after curing persist clinically. Fifth, optical properties were outside the scope of this evaluation. Translucency and light transmittance are not reported here, so the association between optical behavior and polymerization performance could not be tested within this dataset, and the optical explanations offered in the discussion therefore remain hypotheses. Sixth, and related to the previous point, the enamel, body and dentin groups are manufacturer-designated categories. These categories may differ in pigment content, opacifier concentration and filler-related light scattering, and do not necessarily represent a simple, ordered opacity gradient; opacity was consequently analyzed as a categorical factor and not as a quantitatively measured independent variable. Seventh, the sample size was calculated to detect a large interaction effect (*f*=0.50), so the study was not designed to detect smaller interactions; the absence of a statistically significant opacity category × curing distance interaction should therefore be read as a failure to detect one at this sample size rather than as evidence that the two factors act independently. Finally, a standardized 20 s exposure was applied to all groups to permit direct comparison, although the manufacturer recommends 30 s for the dentin opacity. Future work should determine whether manufacturer-specific protocols or longer irradiation can compensate for the reduced hardness-based curing outcomes observed for the body and dentin categories, and should combine direct optical characterization with degree-of-conversion measurement in a range of APS-based materials.

## Conclusions

Within the limitations of this *in vitro* study, manufacturer-designated opacity category significantly influenced the hardness-based curing outcomes of the tested APS-based nanohybrid resin composite. Enamel WE showed the highest bottom-to-top hardness ratio and was the only category to exceed the 80% criterion at both curing distances, whereas body A1 and dentin DA1 remained below this threshold, with dentin DA1 showing the lowest bottom surface microhardness and hardness ratio. Increasing the curing distance from 0 to 2 mm significantly reduced bottom surface microhardness but did not significantly affect top surface microhardness or the overall bottom-to-top hardness ratio, and no significant opacity category × curing distance interaction was detected. Because the optical properties of the tested categories were not quantified, the observed differences cannot be directly attributed to translucency or other optical characteristics. Clinically, these findings suggest that the manufacturer-designated opacity categories of this particular APS-based composite should not be assumed to exhibit equivalent curing behavior under an identical light-curing protocol. These findings are material-specific and should not be generalized to APS-based composites as a class. 

## Figures and Tables

**Table 1:** Table 1: Material characteristics, manufacturer-designated opacity categories, polymerization technology and lot numbers of the APS-based nanohybrid resin composite.

**Composite**	**Opacity category**	**Manufacturer**	**Filler content**	**Filler type**	**Polymerization technology**	**Lot no.**
Elora APS	Enamel Effect / WE	FGM, Joinville-SC, Brazil	Barium glass, spherical zirconia silicate, spherical synthetic silica; amorphous synthetic silica (>76 wt%)	Nanohybrid	APS (Advanced Polymerization System)	090725
	Body / A1					220825
	Dentin / DA1					090725

**Table 2:** Table 2: Top surface microhardness, bottom surface microhardness and bottom-to-top hardness ratio of the APS-based nanohybrid resin composite according to manufacturer-designated opacity category and curing distance (mean ± SD and 95% CI).

**Opacity category**	**Curing distance**	**Top surface VHN (95% CI)**	**Bottom surface VHN (95% CI)**	**Hardness ratio % (95% CI)**
Enamel (WE)	0 mm	80.08 ± 1.66 A,a (78.54-81.61)	70.92 ± 2.09 A,a (68.99-72.85)	88.61 ± 3.60 A,a (85.28-91.94)
Enamel (WE)	2 mm	78.33 ± 2.12 A,a (76.38-80.29)	70.02 ± 2.82 A,a (67.42-72.63)	89.47 ± 4.82 A,a (85.01-93.92)
Body (A1)	0 mm	90.08 ± 2.39 A,b (87.87-92.29)	71.75 ± 4.50 A,a (67.59-75.91)	79.70 ± 5.41 A,b (74.69-84.71)
Body (A1)	2 mm	89.95 ± 2.24 A,b (87.87-92.02)	66.41 ± 2.10 B,a (64.48-68.35)	73.88 ± 3.17 B,b (70.95-76.82)
Dentin (DA1)	0 mm	90.67 ± 2.25 A,b (88.59-92.76)	61.03 ± 3.94 A,b (57.39-64.68)	67.32 ± 4.26 A,c (63.38-71.26)
Dentin (DA1)	2 mm	88.92 ± 1.88 A,b (87.18-90.66)	57.85 ± 3.02 A,b (55.05-60.64)	65.10 ± 4.01 A,c (61.39-68.80)

VHN: Vickers hardness number; CI: Confidence interval. Hardness ratio = (VHNb_ottom_ / VHN_top_) × 100. Different lowercase letters indicate significant differences among manufacturer-designated opacity categories within the same curing distance (Tukey HSD, p<0.05). Different uppercase letters indicate significant differences between curing distances within the same opacity category (pairwise contrasts based on the two-way ANOVA error term, p<0.05).

**Table 3:** Table 3: Two-way ANOVA for the effects of opacity category and curing distance on top surface microhardness, bottom surface microhardness and the bottom-to-top hardness ratio.

**Outcome**	**Source of variation**	**Type III SS**	**df**	**MS**	**F**	*p*	**ηp²**
Top surface VHN	Opacity category	1.068.885	2	534.442	120.656	<0.001*	0.870
	Curing distance	15.357	1	15.357	3.467	0.071	0.088
	Opacity category × curing distance	6.084	2	3.042	0.687	0.510	0.037
	Error	159.461	36	4.429	-	-	-
Bottom surface VHN	Opacity category	1.010.769	2	505.385	49.214	<0.001*	0.732
	Curing distance	103.505	1	103.505	10.079	0.003*	0.219
	Opacity category × curing distance	34.554	2	17.277	1.682	0.200	0.085
	Error	369.687	36	10.269	-	-	-
Hardness ratio (%)	Opacity category	3.654.917	2	1.827.459	99.886	<0.001*	0.847
	Curing distance	60.259	1	60.259	3.294	0.078	0.084
	Opacity category × curing distance	78.162	2	39.081	2.136	0.133	0.106
	Error	658.634	36	18.295	-	-	-

SS: Sum of squares; df: Degrees of freedom; MS: Mean square; ηp^2^: Partial eta squared. R^2^=0.872 (adjusted 0.855) for top surface microhardness, 0.757 (adjusted 0.723) for bottom surface microhardness and 0.852 (adjusted 0.832) for the hardness ratio. *Significant at p<0.05.

**Figure 1:**
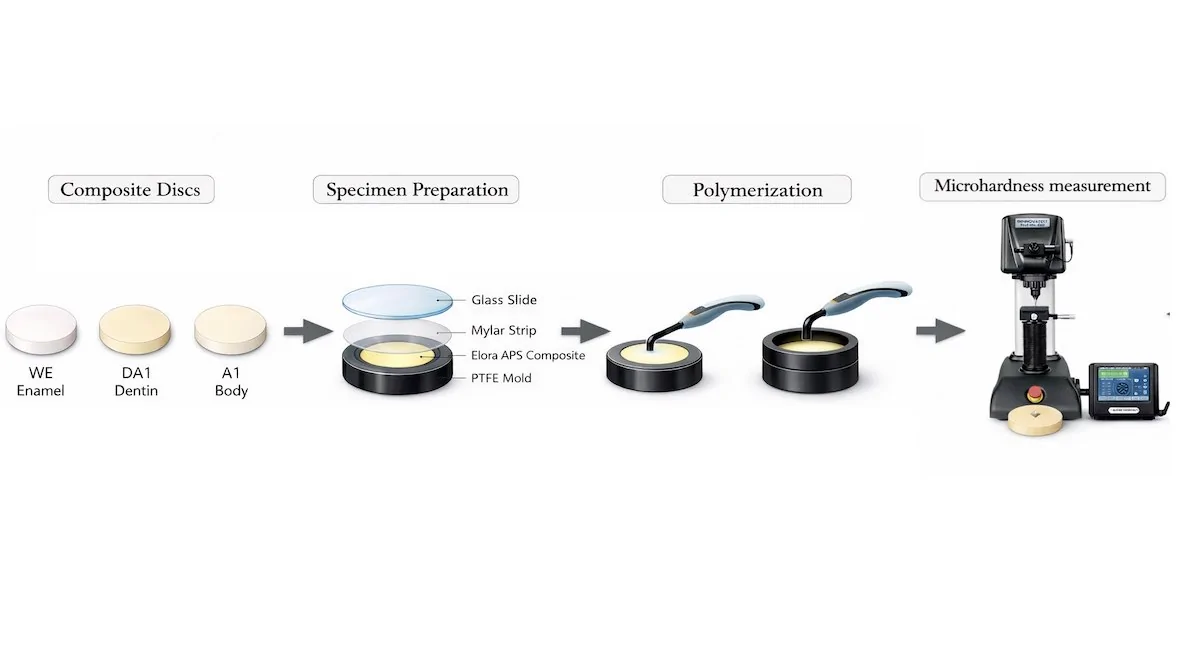
Fig. 1: Schematic overview of composite specimen preparation, light-curing procedures and subsequent microhardness measurements.

## Data Availability

The data that support the findings of this study are available from the corresponding author upon reasonable request.
